# Diagnostic Pitfall in Atypical Febrile Presentation in a Patient with a Pregnancy-Specific Dermatosis—Case Report and Literature Review

**DOI:** 10.3390/medicina58070847

**Published:** 2022-06-25

**Authors:** Claudia Mehedintu, Florin Isopescu, Oana-Maria Ionescu, Aida Petca, Elvira Bratila, Monica Mihaela Cirstoiu, Andreea Carp-Veliscu, Francesca Frincu

**Affiliations:** Department of Obstetrics and Gynecology, “Carol Davila” University of Medicine and Pharmacy, 020021 Bucharest, Romania; claudia.mehedintu@umfcd.ro (C.M.); ionescuoanamaria@gmail.com (O.-M.I.); aida.petca@umfcd.ro (A.P.); elvira.bratila@umfcd.ro (E.B.); monica.cirstoiu@umfcd.ro (M.M.C.); andreea_veliscu@yahoo.com (A.C.-V.); francesca.frincu@drd.umfcd.ro (F.F.)

**Keywords:** PUPPP, dermatoses, fever, pregnancy, skin, pruritus, urticaria

## Abstract

Pruritic urticarial papules and plaques of pregnancy (PUPPP) usually occurs in the third trimester of pregnancy in primiparous women. It is a self-limiting inflammatory disorder with a still unknown pathogenic mechanism. The abdominal wall overdistension, with a subsequent inflammatory response due to damage to the connective tissue, represents a pathogenesis explanation. Clinical features involve intensely pruritic urticarial rash with edematous, erythematous papules and plaques. The clinical picture and dermal biopsy establish the diagnosis. Topical corticosteroids and oral antihistamines are usually sufficient, but sometimes systemic corticosteroids are necessary. Maternal and fetal prognosis is excellent, and the lesions resolve after birth with no scarring or pigmentary change. We present a case of a 36-year-old patient with a 32-week pregnancy who was admitted with a generalized pruritic rash accompanied by fever. The final diagnosis was decided after multiple pathology exclusions. Treatment consisted of systemic corticoid therapy. The patient gave birth by cesarean section to a healthy newborn without dermatological lesions or other conditions. Adding more PUPPP cases to the literature portfolio will bring more awareness to this under-recognized and under-reported skin disorder. We trust this case will encourage other physicians to publish more cases of pregnancy-specific dermatoses.

## 1. Introduction

First described by Lawley et al. as an intensely pruritic cutaneous eruption in the third trimester of pregnancy [1], pruritic urticarial papules and plaques of pregnancy (PUPPP) is a benign, inflammatory skin disorder with a pruritic character that usually affects primigravidae in their late stage of pregnancy [2]. It is also known as polymorphic eruption of pregnancy (PEP) and belongs to a group of specific dermatoses that appear only in the pregnant or puerperal state [3]. The parity (primiparous) and late-third-trimester onset lend support to the pathogenic hypothesis of the overdistension of the abdominal wall, also known as the “theory of distension”, suggesting that such overdistension may cause injury to the connective tissue within the striae gravidarum with antigenic molecules generation and inflammatory response [4]. Despite these observations, the pathogenic mechanisms of PUPPP are still not fully understood [5]. The inconsistent clinical presentation, the rarity of this condition, and the lack of specific laboratory tests may lead to confusion when establishing the diagnosis and appropriate treatment. This paper presents a case of a 36-year-old patient with a 32-week pregnancy who was admitted with a generalized pruritic rash accompanied by fever. To our knowledge, this is the first case of PUPPP associated with pyrexia. This additional clinical sign challenged our diagnosing strategy, as we were forced to delay treatment and exclude multiple pathologies. We trust that this case presentation together with the literature review will bring more awareness regarding an under-recognized and under-reported skin disorder in pregnancy.

## 2. Case Presentation

**Figure 1:**
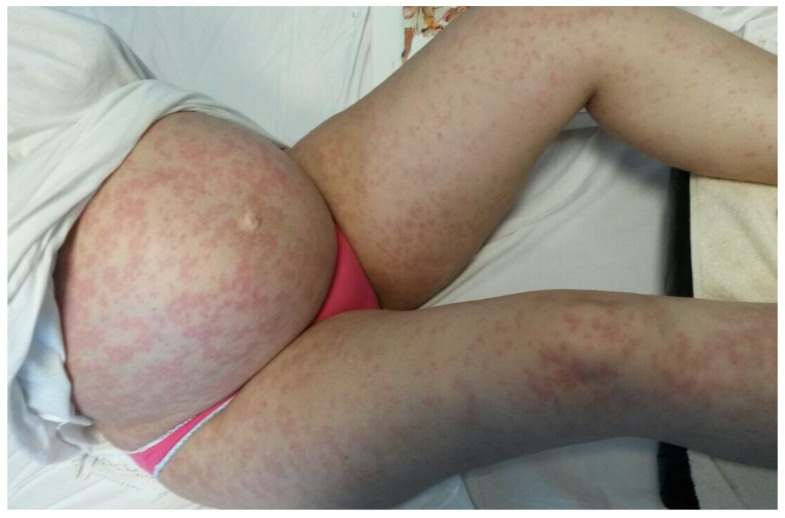
A monomorphic rash consisting of erythematous papules and round oval plaques disseminated on the abdomen and lower limbs, that spares the umbilicus.

**Figure 2:**
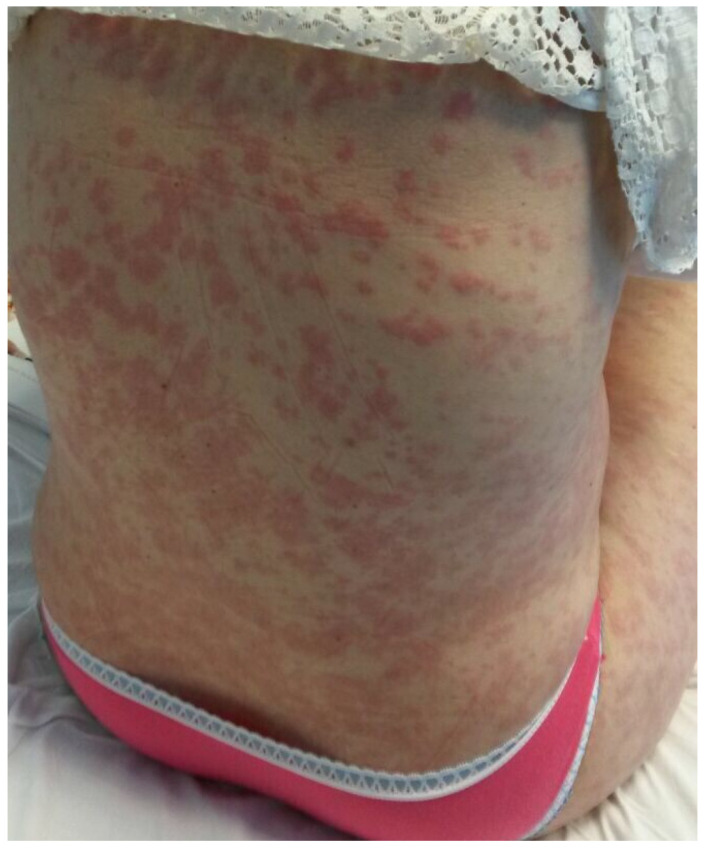
Erythematous papules and plaques on the trunk and inferior limbs. Lesions vary in size from 0.5 cm to large confluent areas.

**Figure 3:**
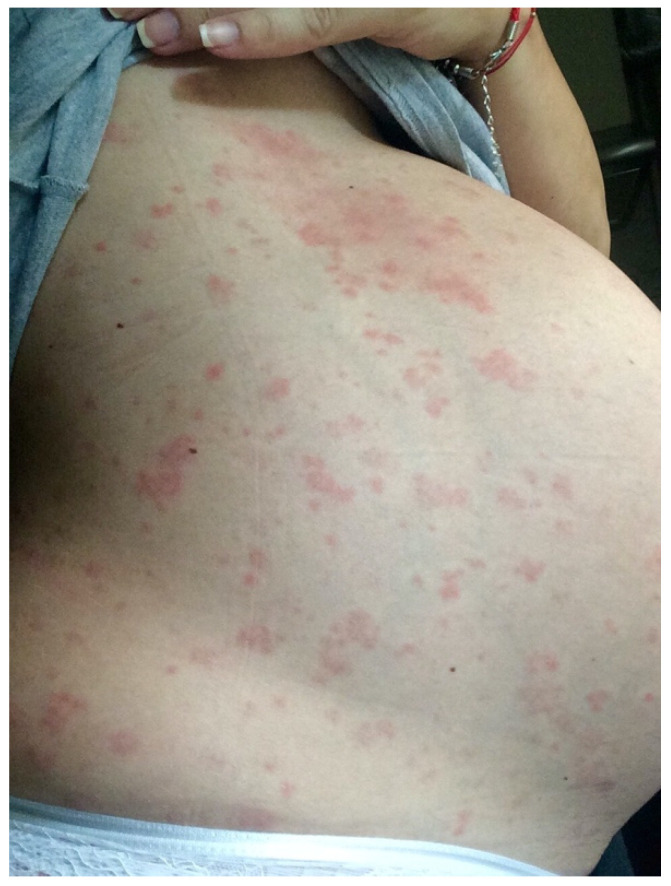
Close representation of the papules and plaques on the abdomen.

**Figure 4:**
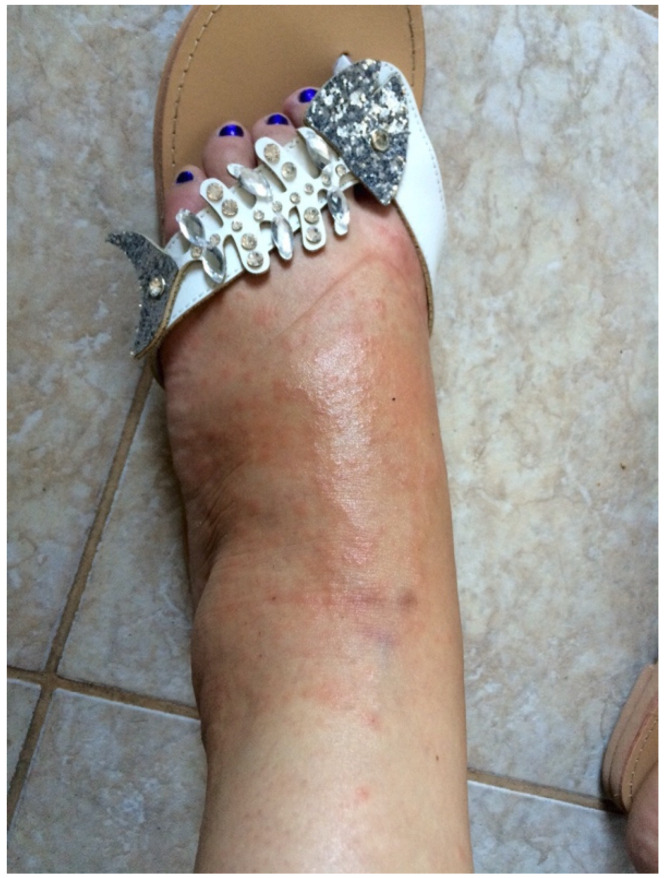
Confluent papules present on the feet.

A 36-year-old, 32-week primiparous pregnant woman was referred to our service with an extensive pruritic rash that initially appeared as erythematous papules on the abdomen. They had rapidly spread to the trunk, thighs, buttocks, calves, legs, and arms, being unresponsive to topical corticosteroids (Figure 1, Figure 2, Figure 3 and Figure 4). On admission, clinical examination showed a body temperature of 38 °C. The patient denied other illnesses and had a weight gain of 15 kg during the first two trimesters of pregnancy.

Ultrasound evaluation showed a 32-week fetus with normal morphology, with an estimated weight of 2350 g (above 90th percentile), and slightly tachycardic (fetal heart rate: 180–200 bpm). The amniotic fluid index (AFI) was within the normal range. All paraclinical investigations were within normal limits, including additional immune tests for anti-neutrophil cytoplasmic antibodies (c-ANCA), perinuclear ANCA (*p*-ANCA), circulating immune complexes, and rheumatoid factor. Due to fever persistence, the patient was transferred to an infectious diseases ward, where an infectious cause was excluded. Virology and parasitology tests revealed no active infection with rubella virus, enteric cytopathic human orphan (ECHO) virus, coxsackievirus, cytomegalovirus, Epstein-Barr virus, herpes simplex virus 1 and 2, HIV 1 and 2, and toxoplasma gondii. Procalcitonin and c-reactive protein were negative. Hematological consultation was also requested, and tests were negative for possible leukemia and lymphoma. Pemphigoid gestationis was initially excluded based on negative ELISA (anti BP-180 antibodies: 12 U/mL; normal values < 20 U/mL) and afterwards by indirect immunofluorescence, with negative anti BP-180 and anti BP-230 antibodies, and normal leukocyte formula. The patient denied skin biopsy; therefore, a PUPPP diagnosis by exclusion was established following an interdisciplinary approach with a dermatologist.

**Figure 5:**
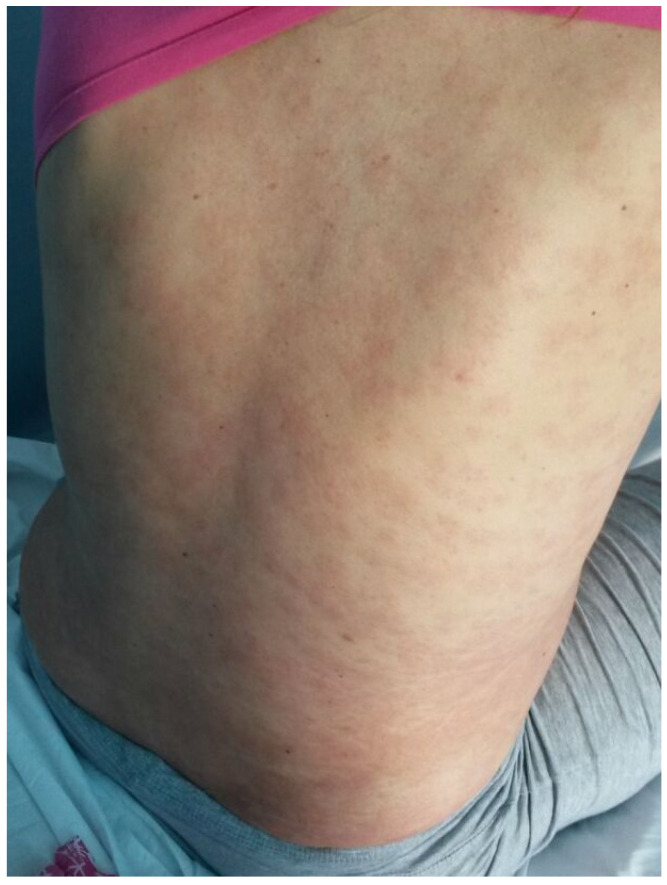
Residual erythematous patches after systemic corticosteroid treatment.

The patient returned to the obstetrics-gynecology ward. Upon admission, she received intravenous hydration. Because of the extent of the lesions and failure of the topical steroid treatment, intravenous treatment with high doses of hydrocortisone hemisuccinate was initiated (100 mg every 6 h, for 2 days). It continued with 200 mg per day (100 mg every 12 h) for 4 days, and progressively decreased to 100 mg per day. The fever subsided from the first day of treatment and did not relapse. Fetal tachycardia remitted in less than 6 h, and the rash gradually remitted under the corticosteroid treatment (Figure 5). During hospitalization, the fetus’s well-being was carefully monitored, with no signs of fetal distress detected.

The patient was discharged after 12 days and had an uneventful pregnancy course upon 36 weeks of gestation, when she gave birth via cesarean section to a 2650 g baby girl because of premature membrane rupture. The maternal dermatological condition did not influence the onset of birth. The evolution of the newborn was favorable, and the postnatal adaptation was appropriate, without dermatological lesions or other diseases. At 3-month follow-up, the patient did not report any skin lesion.

## 3. Materials and Method

PubMed and EMBASE were searched from their inception until 15 May 2022 for all studies on pruritic papules and plaques of pregnancy or polymorphic eruption of pregnancy. We focused our search on pathogenic mechanisms, incidence, immunohistochemistry, immunology, classifications, and treatment. The reference lists of the included studies were also screened for additional literature. A total of 949 cases of PUPPP/PEP were identified from reported case reports, case series, case-control studies, and retrospective and prospective studies.

We used “MeSH” (PubMed) and “Emtree” (EMBASE) terms, but also free text words. To be as comprehensive as possible, the search was not restricted to any study types for the review section. Studies in languages other than English were excluded.

The clinical case publication procedure was approved by the Local Ethics Committee (Registry Nr. 70/24.02.2022). The patient provided her written informed consent for publication.

## 4. Discussion

Pregnancy is a predisposing condition for benign skin changes that may occur because of hormonal/physiological changes or immune-hormonal alterations, either de novo or through amplification of pre-existing lesions that may manifest a flare-up [6]. First named as erythema multiforme of pregnancy, then as toxemic rash of pregnancy, late onset prurigo of pregnancy, PUPPP was redefined in 1979 by Lawley et al. [1] based on biopsies. In 1983, Holmes and Black [7] introduced a synonym for PUPPP, most used outside of the United States, namely polymorphic eruption of pregnancy (PEP) [7]. This nomenclature was introduced because of the broad variety of lesion morphologies, which may include eczematous lesions with excoriated papules, plaques, desquamation, and crusts; vesicles; non-urticarial polycyclic erythema; and targetoid or erythema-multiforme-like lesions [8,9]. Aronson et al. [10] further subclassified PUPPP into three clinical categories: type I PUPPP describes the urticarial papules and plaques without excoriations and vesicles; type II PUPPP has predominantly non-urticarial erythema, with variable vesicles, excoriations, and 1–2 mm erythematous papules (maculopapular type); and type III PUPPP shows a combination of type I and type II.

PUPPP is a self-limited pruritic inflammatory skin disorder of pregnancy [11]. The lesion distribution at onset is within the abdominal striae, with a later spread within days to arms, thighs, and breasts. Periumbilical sparing is typical and helps differentiate PUPPP from other gestational dermatoses, such as pemphigoid gestationis (PG), pruritic folliculitis of pregnancy, and prurigo of pregnancy [10,12,13]. Small erythematous papules with itchy character can further coalesce and form greater urticarial plaques on the abdominal area, surrounded by whitish halos [5,14,15]. PUPPP lesions rarely occur on the face, palms, or soles [3]. Ghazeeri et al. [16] reported two cases that had an eruption onset on the lower extremities with rapid lesion spreading to the abdomen, and another four cases with exclusive involvement of the peripheral limbs. Carruthers et al. [17], Alcalay et al. [18], and Kirkup and Dunhill [19] reported facial distribution in PUPPP. PUPPP lesions on the soles and palms may appear with micro-vesiculation, even with pompholyx presentation, which makes it harder to differentiate from PG [13]. Vaughan Jones et al. [20] also reported the Koebner phenomenon in previous scars. As the disease advances, the morphology changes with possible development of annular wheals, papulovesicles, erythema, and targetoid lesions, assigning a polymorphic character [5,21]. The mucous membranes are not affected in PUPPP [5].

PUPPP actual incidence is not known, but one out of 130–300 pregnancies have been reported [22], and Elling et al. [23] reported 1 in 200 single pregnancies (0.5%). Literature findings suggest that multiple pregnancies carry a greater risk of developing this skin condition, with an incidence of 2.9–16% in twin pregnancies and 14–17% in triplets [24]. PUPPP usually appears among primigravidae in the third trimester of pregnancy (with a mean onset of 35 weeks of gestation) or during the immediate postpartum period, with no tendency to relapse in subsequent pregnancies [21,22,25]. Ghazeeri et al. [16] found a link between Rh-positive mothers and this disease and reported that 89% of cases with PUPPP were obtained through in vitro conception [16]. Other risk factors for PUPPP include hydramnios and fetal macrosomia (two causes of overdistension of the abdominal skin in pregnancy) and male fetus [11,26,27]. In our case, the patient’s weight gain on admission was 15 kg, and she delivered a 2650 g female fetus (above 95th percentile).

Regarding the etiology of PUPPP, the literature is deficient because of under-reported cases and the lack of randomized controlled studies. Over time, several authors have assumed and performed numerous tests to investigate the cause of this pathology. Hormonal factors, abdominal distention, fetal DNA from maternal skin lesions, and placental factors have been the most incriminated, but a final decision on the exact pathogenesis of PUPPP could not be drawn [13]. Multiple immunological and infectious assays were performed in sporadically reported cases. The rapid stretching of the abdominal skin may lead to connective tissue damage, exposing the collagen to newly formed antigenic molecules. An allergic-type response could be, therefore, induced, leading to the eruption in striae [5,12]. Excessive abdominal distention is directly proportionally associated with multiple gestation pregnancies, and multiple gestation is linked with higher progesterone and estrogen levels [28,29,30]. The inflammatory process at the skin level is worsened by progesterone, and PUPPP lesions showed increased immunoreactivity of the progesterone receptors [12]. A study by Panicker et al. [6] showed that purple striae gravidarum are more common among primigravidae (45.67%) and appear from the second trimester, whereas atrophic striae are common among multigravidae (76.95%) [6]. Postpartum PUPPP may be related to the substantial skin stretching during the third trimester, followed by the rapid decrease in skin stretching after delivery [14,31]. A delayed hypersensitivity reaction to an undetermined antigen may be involved, as immune tolerance during subsequent pregnancies might explain the non-recurrent character [5,12,32]. Some authors speculated that the third-trimester-aged placenta determines maternal fibroblast proliferation through released molecules known as F-substance [22,33]. Dominguez-Serrano et al. [24] assumed a greater body mass index (BMI) could be associated with a higher risk of PUPPP [24]. Similarly, Ghazeeri et al. [16] affirmed that 75% of PUPPP patients presented excessive maternal weight gain. Moreover, Vaughan Jones et al. [20] showed that the cortisol level was reduced in pregnant women with PUPPP (*p* = 0.03), whereas beta-hCG, androgens, and estradiol were similar to the control group. Aractingi et al. [34] were among the first to demonstrate the presence of fetal DNA in the maternal dermis, leading to a new etiopathogenic theory. In the same manner, Nelson et al. [35] and Matz et al. [36] pointed the microchimerism between peripheral blood and pregnancy as a trigger for the fetal cells to migrate to the maternal dermis and generate the eruption. The source of fetal DNA is thought to be fetal lymphocytes or trophoblastic cells [12]. Recently, Ishikawa-Nishimura et al. [37], but also Matsumoto [38], assumed that skin-resident bacteria and fungi, such as staphylococcus aureus, malassezia, and candida, invade the skin through eczematous lesions and upregulate the Th2 cytokine profile, generating IL-9 and IL-33, which are usually undetectable in normal pregnancy. Moreover, Rudolph et al. [13] showed that 55% of patients with PUPPP also had a history of atopy, suggesting a link between the two conditions, especially in those with longer disease duration, and concluded that atopy prolongs and worsen PUPPP symptoms.

Most cases are clinically diagnosed without the need for invasive testing [11]. However, when skin biopsies are performed to differentiate PUPPP from other pregnancy dermatoses (in cases with no response to topic corticoid treatment or in doubt), histopathology specimens from PUPPP lesions show nonspecific findings. In early PUPPP lesions, epidermal and upper papillary dermal edema can be observed, as well as focal mild spongiosis, with a lymphocytic perivascular infiltrate consisting of T-helper lymphocytes mixed with neutrophils and eosinophils in the deeper dermis, resembling arthropod bite reactions [5,7,16]. During the convalescent phase of the disease, hyperkeratosis and parakeratosis may be observed. While indirect immunofluorescence (IIF) is always negative for circulating immunoglobin G (IgG) autoantibodies to the basement membrane, direct immunofluorescence (DIF) occasionally reported deposits of complement 3 (C3) and IgM in vessel walls and granular deposits of C3 within the dermo-epidermal junction (DEJ) [3,8,20]. C3 accumulation proved to be a sign of excoriation (20). In contrast, Zurn et al. [39] reported only five in 106 cases (4.5%) of circulating anti-basement membrane zone (BMZ) IgM at IIF. However, this is the only report in the literature, and when it comes to differentiating PUPPP from pemphigoid gestationis, IIF is the most frequently considered [16]. From the above, we can conclude that the phenotypic manifestations vary greatly, and we must be highly oriented towards a differential diagnosis.

In 2006, Ambrus-Rudolph [9] proposed a new classification as follows: polymorphic eruption of pregnancy (PEP), pemphigoid gestationis (PG), the atopic eruption of pregnancy (AEP), and intrahepatic cholestasis of pregnancy (ICP) [8]. More recently, Danesh et al. [9] re-added in 2016 a fifth dermatosis to the classification, namely pustular psoriasis of pregnancy (PPP) [9]. Several revisions of the classification and nomenclature, along with misnomers, modern and historical, generated confusion. Gestational prurigo is characterized by earlier onset in pregnancy and persistence after birth of 1–5 mm erythematous papules on extensor surfaces and trunk [16]. Eczema of pregnancy develops as erythematous, scaly macules on the flexural folds of the extremities, neck, and face in patients with a family history of atopic terrain [5]. The clinical picture with the abovementioned features, together with the biopsy and negative direct and indirect immunofluorescence, establish the diagnosis [40]. An unusual early onset is considered before 35 weeks’ gestation, and prolonged disease duration is more than 6 weeks. Multigravida and multiple gestation pregnancies are associated early onset of the condition, whereas atopy is related to longer duration [13,20]. PG is characterized by pruritic papules and urticarial plaques that are later complicated with vesicles and bullae and involve the tegument around the umbilical region [41]. It affects women in early pregnancy stages and is associated with fetal adverse effects: intrauterine growth restriction, premature birth, and placental insufficiency through IgG and C3 deposition in the amniotic membrane [42]. DIF shows IgG and C3 deposits within DEJ and helps reach the final diagnosis [43]. Apart from PG, toxic drug eruption and allergic drug reactions (most commonly to antibiotics and non-steroidal anti-inflammatory drugs), urticaria, contact dermatitis, scabies, and viral exanthema need to be ruled out [13,17,40,44].

Literature research does not report an association between fever and PUPPP. Kanj R. et al. [45] presented a case report where a systemic anaplastic large cell lymphoma, with cerebral and lung nodules, presenting skin involvement was misdiagnosed as PUPPP [45]. It is important to consider hematological conditions in pregnant women with cutaneous lesions. In our case, the onset of fever was idiopathic. No connection with another pathology of infectious cause could be established. We assume it was associated with PUPPP, as it remitted with corticosteroids.

Although PUPPP is a self-limited condition, it requires symptomatic treatment in most cases. The pruritus can reach an intractable and severe level, but the PUPPP outcome is favorable for both mother and fetus [22]. Bland emollients with first-generation oral antihistamines and a moderately potent topical corticosteroid may help [5,20,32]. In some cases, the pruritus can become intractable, with sleep disturbances; therefore, systemic corticosteroids may be needful [3,16]. Non-halogenated glucocorticosteroids, such as prednisolone, are preferable in severe cases because of enzymatic inactivation in the placenta and should be administered in a dosage of 0.5–2 mg/kg/day [46]. Prednisolone in a dose of 40 mg to 60 mg induces a rapid remission of symptoms, and the prednisolone gradient in mother-fetal blood is only 10:1 [47,48]. Cold baths, applications of menthol or urea, and wearing cotton clothing are also helpful in reducing symptoms [5,20]. Scheinfeld [49] proposed fluticasone propionate 0.005 percent lotion, a class 5 (low-medium potency) corticosteroid, to be considered for the treatment of PUPPP. PUPPP does not represent an indication for early delivery [20], since this self-limited condition poses no risk for the mother or fetus [50]. However, Beltrani et al. [51] performed a cesarean section at 35 weeks’ gestation on a patient with severe PUPPP, irresponsive to treatment, and symptoms improved 12 h postpartum. The lesions usually tend to remit spontaneously within 7 to 10 days postpartum, and the duration of the rash can vary from 4 to 6 weeks, without scarring or pigmentary changes [10,13,22]. New treatment options, such as autologous whole blood (AWB) injection, often used for chronic urticaria and atopic dermatitis, proved useful in PUPPP treatment [14]. It appears that AWB may modulate the maternal immune system regarding disease development [52]. Unlike other pregnancy-specific dermatoses, such as gestational pemphigoid and intrahepatic cholestasis of pregnancy, the maternal-fetal prognosis is not altered in PUPPP [3,53]. In contrast to most literature reports, sporadic cases of pregnancy-induced hypertension with PUPPP have been described. Ohel et al. [54] a found a significant association between the two conditions, but Regnier et al. [55] only reported a higher risk with no statistical significance. No intrauterine growth restriction or lesion tegmental changes have been observed in newborns [10,13].

## 5. Conclusions

In conclusion, PUPPP belongs to a group of pregnancy-specific dermatoses of yet unknown etiology, affecting mainly primiparous women in the third trimester of pregnancy, with eruptions most often affecting the abdomen, buttocks, and lower limbs. Fever is not a specific PUPPP symptom, but when it occurs, infectious and hematological causes must be excluded. Topical conservative treatment to reduce symptoms is effective, and only refractory cases require systemic corticosteroids. The course is benign, with no adverse effects on pregnancy or labor, and normal fetal development, with spontaneous resolution within a few days after birth. This case presentation aims to add an atypical PUPPP case to the literature portfolio and to encourage physicians to publish more cases of pregnancy-specific dermatoses.

## Data Availability

All the data are available from the corresponding author upon reasonable request.
